# Mutations within the miR172 target site of wheat *AP2* homoeologs regulate lodicule size and rachis internode length

**DOI:** 10.1270/jsbbs.23019

**Published:** 2023-09-09

**Authors:** Agetha Bigie Nanape, Hlaing Moe Haine, Kazuhiko Sugimoto, Fuminori Kobayashi, Youko Oono, Hirokazu Handa, Takao Komatsuda, Katsuyuki Kakeda

**Affiliations:** 1 Graduate School of Bioresources, Mie University, 1577 Kurimamachiya-cho, Tsu, Mie 514-8507, Japan; 2 Institute of Crop Science, National Agriculture and Food Research Organization (NARO), 2-1-2 Kannondai, Tsukuba, Ibaraki 305-8518, Japan; 3 Graduate School of Life and Environmental Sciences, Kyoto Prefectural University, 1-5 Shimogamohangi-cho, Sakyo-ku, Kyoto 606-8522, Japan; 4 Shandong Academy of Agricultural Sciences (SAAS), Crop Research Institute, 202 Gongyebei Road, Licheng District, Jinan, Shandong 250100, China

**Keywords:** *Triticum aestivum*, *Apetala2*, microRNA172, SNP, cleistogamy

## Abstract

Closed fertilization in flowers, or cleistogamy, reduces the risk of fungal infection in Triticeae crops. In barley (*Hordeum vulgare*), cleistogamy is determined by a single recessive gene, *cly1*, which results from a single nucleotide polymorphism within the microRNA172 target site of the *Apetala2* (*AP2*) transcription factor gene. The recessive *cly1* allele negatively regulates the development of lodicules, keeping florets closed at anthesis. However, cleistogamy is not evident in hexaploid wheat (*Triticum aestivum*) cultivars. This study aimed at identifying mutations in wheat *AP2* orthologs by ethyl methane sulfonate–induced mutagenesis and high-resolution melt analysis. Although flowers of *AP2* mutants induced in the A and D genomes opened at anthesis, their lodicule size was significantly smaller, especially in the direction of depth, than that of wild-type plants. One of the mutants that carried a nucleotide replacement in *AP2* from the D genome produced a compact spike caused by a substantial decrease in rachis internode length, analogous to the barley dense spike. Cleistogamous hexaploid wheat might be generated by combining effective mutant alleles of *AP2*-homoeologous genes.

## Introduction

Flowers in many grass species open as the swelling lodicules push apart the lemma and palea at anthesis. Open flowering, called chasmogamy, is the primary type of flowering in wheat (*Triticum aestivum*), barley (*Hordeum vulgare*), and other species. Some cultivars of barley, however, flower by cleistogamy (closed flowering at pollination/fertilization). In cleistogamous flowering, the lodicules are rudimentary and do not swell, so the floret remains closed. Cleistogamy in barley is determined by a single gene at the *Cleistogamy 1* (*cly1*) locus on chromosome 2H (Turuspekov *et al.* 2004). The *Cly1* gene encodes HvAP2, a barley ortholog of the *Arabidopsis* Apetala2 (AP2) transcription factor protein, and *Cly1* mRNA contains a microRNA172 (miR172) target site in the 10th exon (Nair *et al.* 2010). Cleistogamous barley is homozygous for one of two recessive *cly1* alleles (*cly1.b*, *cly1.c*), which are natural variants each carrying a synonymous single nucleotide polymorphism (SNP) within the miR172 target site. The nucleotide change inhibits the binding of miR172 to the target site of *Cly1* mRNA in the lodicule and leads to production of the HvAP2 protein, which is assumed to negatively regulate lodicule development (Anwar *et al.* 2018).

Cleistogamous barley cultivars have been used in breeding programs, consciously or unconsciously, as they offer the advantage of evading infection by the fungus causing Fusarium head blight (FHB). Tests using spray inoculation with *Fusarium graminearum* showed a clear difference in FHB severity between cleistogamous and chasmogamous barley lines, and revealed the greater contribution of cleistogamy to FHB resistance than of row type and other tested spike characters (Yoshida *et al.* 2005). A survey of the effect of infection timing also indicated that cleistogamous barley cultivars were resistant at anthesis but susceptible 10 days later, whereas chasmogamous cultivars were already susceptible at anthesis (Yoshida *et al.* 2007). On the basis of these findings in barley, Ning *et al.* (2013a) attempted to isolate wheat orthologs of the barley *Cly1* (*HvAP2*) gene as rational targets for engineering cleistogamy in wheat, and successfully cloned three *AP2* homoeologs (designated *AP2-A*, *-B*, and *-D*) from hexaploid wheat. Natural sequence variations within their miR172 target site have been sought in a wide range of diploid, tetraploid, and hexaploid wheat accessions. However, the sequences of wheat *AP2* homoeologs are highly conserved across ploidy levels, and no sequence variant within the miR172 target site has been detected in any homoeolog (Ning *et al.* 2013b). The aim of this study was to isolate mutants carrying a novel point mutation within the miR172 target site in wheat *AP2* homoeologs. Here, we identified such point mutations in mutants induced by chemical mutagenesis, and analyzed the lodicule and other spike traits of the mutants.

## Materials and Methods

### Plant materials

The *AP2* mutants analyzed in this study were induced from the Japanese winter wheat cultivar ‘Kitahonami’. Mutants carrying a point mutation within the miR172 target site were screened among 2157 M_2_ individuals generated by single-seed descent from M_1_ plants obtained from seeds treated with 0.5%, 0.7% or 0.75% ethyl methanesulfonate (EMS) solution for 18 h. Mutant plants were isolated by high-resolution melt (HRM) analysis, described next. In addition, null (deletion) mutants were screened from another M_2_ population consisting of 1440 individuals that were generated by gamma-irradiation at 250 Gy, as described in Komura *et al.* (2022). Gene-specific PCR was used for each *AP2* homoeolog. All mutant lines of interest were maintained by selfing and the selection of good-fertility plants with no apparent growth defects. M_4_ and M_5_ plants were grown in the field for trait and expression analysis.

### Screening of mutants and molecular analysis with genomic DNA

Genomic DNA was extracted by the CTAB method from the leaves of M_2_ individuals and the parental cultivar. Mutants were screened for SNPs within the miR172 target site by HRM analysis using gene-specific primers for each *AP2* homoeolog (Supplemental Table 1; Ning *et al.* 2013a) in a ViiA 7 system (Applied Biosystems, Tokyo) with a MeltDoctor HRM Reagent Kit (Thermo Fisher Scientific, Tokyo) according to the manufacturers’ instructions. Amplicons of HRM-positive mutants were Sanger-sequenced to identify the positions of the SNPs. For genotyping of M_3_ plants derived from heterozygous M_2_ mutants and for confirming mutants from M_4_ onward, genomic DNA was extracted by the SDS method (Komatsuda *et al.* 1998) and sequenced. A complete DNA sequence (ca. 2.8 kbp) encompassing the coding region of the *AP2-A* or *AP2-D* gene was determined according to Ning *et al.* (2013b). Null mutants were screened by PCR amplification using the same gene-specific primers for each *AP2* homoeolog as described above (Supplemental Table 1; Ning *et al.* 2013a), followed by agarose gel electrophoresis.

### RNA extraction and expression analysis

Total RNA was extracted from immature spikes harvested at the terminal spikelet stage or the white anther stage (Kirby and Appleyard 1981) with an RNeasy Mini Kit (Qiagen, Tokyo) according to Wang *et al.* (2021). Extracted RNA treated with RNase-free DNase I (Qiagen) was used for cDNA synthesis with a High-Capacity RNA-to-cDNA Kit (Thermo Fisher Scientific). Quantitative real-time PCR (qPCR) analysis was performed on a StepOnePlus Real-Time PCR System (Applied Biosystems) with Thunderbird SYBR qPCR Mix (Toyobo, Osaka) according to the manufacturers’ protocols. The analysis used the ΔΔCt method (Livak and Schmittgen 2001) with 3′-UTR primers for each *AP2* homoeolog and the wheat *actin* gene as an internal control (Supplemental Table 1; Ning *et al.* 2013a). Each genotype was assayed as two biological replicates.

### Measurement of lodicule size and spike density

On the day of anthesis, the culm below the flag leaf node was cut and held in water in a test tube at room temperature. Just before anthesis, the first, second or both florets were detached from a spikelet and the lemma was removed. The length, width and depth of each lodicule were measured (Supplemental Fig. 1) under a stereomicroscope with a DFC300 FX digital microscope camera system (Leica Microsystems, Tokyo). About 30 lodicules from 15–27 spikes per genotype were measured.

Using mature (or close to mature) spikes, the rachis length (i.e., the length of the central axis of a spike) was measured and the rachis nodes were counted, and the mean rachis internode length was calculated as an indicator of spike density.

Lodicule size and rachis traits were analyzed by one-way ANOVA, followed by Tukey’s honestly significant difference for multiple comparison (*P* < 0.05).

### Prediction of interaction between miR172 and target mRNA using RNAhybrid

The interaction of miR172 with the target site in the wild-type and mutant alleles of wheat *AP2* homoeologs was predicted by using the RNAhybrid online tool as described in Rehmsmeier *et al.* (2004). RNAhybrid predicts the secondary structure of the miRNA and target mRNA sequences by optimizing their hybridization in terms of minimum free energy (mfe). It extends the classical RNA secondary-structure prediction algorithm and uses the dynamic programming technique to calculate mfe. We used the mature sequence of miR172a for wheat (*TamiR172a*, Ning *et al.* 2013a) and barley (*Hv-miR172a*, Anwar *et al.* 2018) because miR172a in immature spikes is the most abundant of the three isomers (miR172a, b and c) and reduces the abundance of Cly1 (HvAP2) protein in barley (Anwar *et al.* 2018). mRNA sequences from wheat *AP2* alleles along with barley *HvAP2* alleles were used as the targets.

### Data availability statement

All data are available in the manuscript, the supplementary materials, or at publicly accessible repositories (DDBJ accession nos. LC772978 to LC772982).

## Results

### Screening of mutants

Four M_2_ plants carrying a SNP within the miR172 target site were isolated by HRM analysis: 038E (carrying *AP2-A1* as described next), 156E and 514E (*AP2-D1*), and 190E (*AP2-D2*). Plants 038E, 156E, and 514E were heterozygous and 190E was homozygous for the mutation. Although 156E and 514E shared the same SNP, only 514E was selected for analysis because 156E and its progeny showed apparent growth defects. SNPs within the miR172 target site of *AP2-B* were not detected. Gene-specific PCR for each *AP2* homoeolog identified three null mutants, namely 1131A (*AP2-A*), 169B (*AP2-B*), and 248D (*AP2-D*) (Supplemental Fig. 2).

### Identification of novel SNPs within the miR172 target site of wheat AP2 homoeologs

**Fig. 1.**
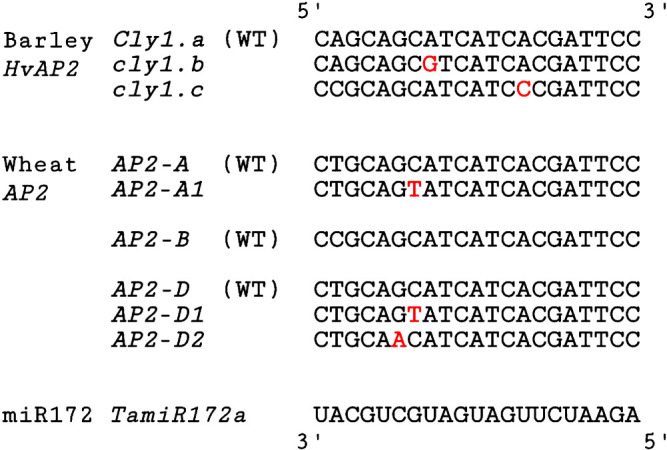
Sequence variation in the miR172 target sites of barley *HvAP2* and wheat *AP2* homoeologs. *AP2-A1*, *D1*, and *D2* are SNP mutant alleles identified among EMS-induced mutants of wild-type (WT) ‘Kitahonami’. The miR172 sequence from wheat *miR172a* is indicated at the bottom.

Three independent novel SNPs within the miR172 target site were identified, and were designated *AP2-A1*, *AP2-D1*, and *AP2-D2* (Fig. 1). The position and nucleotide change of *AP2-A1* and *AP2-D1* were identical within the miR172-targeted 21-nt sequences, with a C-to-T change at the 7th nucleotide position. *AP2-D2* had a G-to-A nucleotide change at the 6th position. These SNPs are distinct from those identified in the cleistogamous alleles *cly1.b* and *cly1.c*, with respective mutations at the 8th and 14th positions (Nair *et al.* 2010) of the orthologous barley gene *Cly1* (Fig. 1). In contrast to the two barley *cly1* alleles each carrying a synonymous SNP, the wheat mutant alleles identified here had non-synonymous SNPs. The translation products of *AP2-A1* and *AP2-D1* would have the Ala residue replaced with Val, while that of *AP2-D2* would have it replaced with Thr. Sequence comparison of PCR-amplified genomic DNA encompassing the coding region (ca. 2.8 kbp) confirmed that all three mutant alleles had no other nucleotide changes from the respective wild-type alleles.

### Phenotypic assessment of mutants

**Fig. 2.**
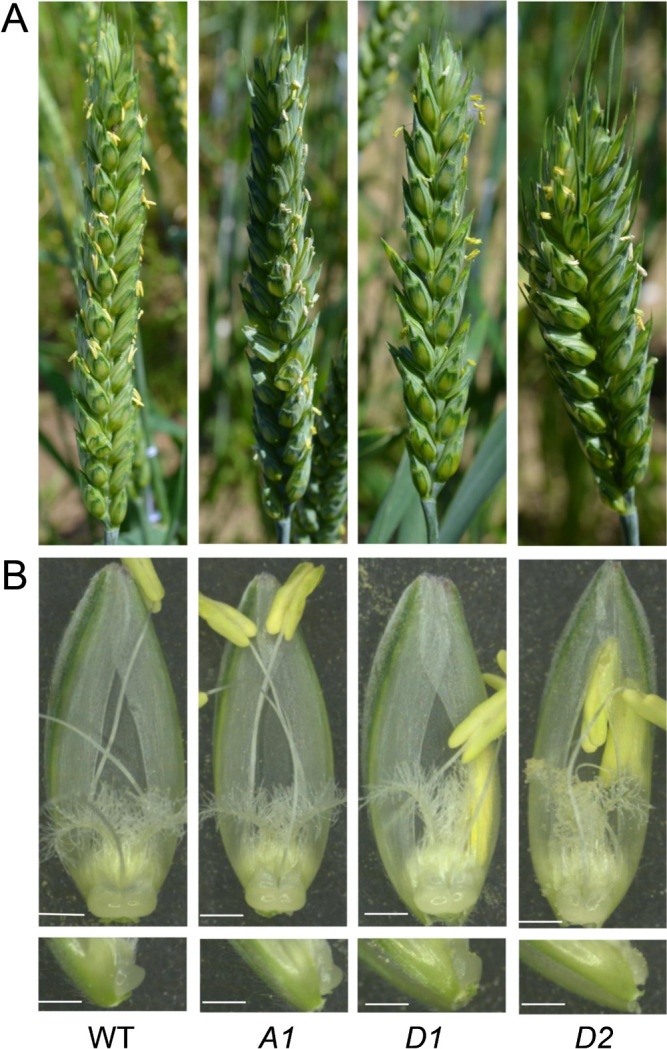
Spikes and floral organs of wild-type (WT) and *AP2* mutants (*A1*, *D1*, *D2*): (A) Spike at anthesis. (B) Floret at anthesis (upper panel) and side view of the lodicule (lower panel). Bar, 1 mm.

**Fig. 3.**
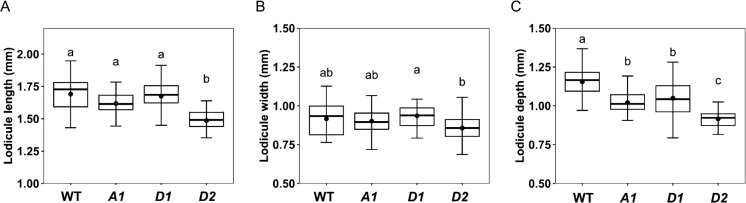
Comparison of lodicule size among wild-type (WT) and *AP2* mutants (*A1*, *D1*, *D2*): (A) Length. (B) Width. (C) Depth. Thick horizontal lines indicate median (50% quartile), bullets indicate mean, and whiskers represent maximum and minimum values excluding outliers. Mean values with the same letter do not differ significantly (*P* > 0.05) by Tukey–Kramer HSD test.

Lines homozygous for the three point mutations grew comparably to the wild type in the field. At anthesis, all mutants flowered with a fairly normal exposure of anthers (Fig. 2A). Lodicules of all mutants swelled at anthesis (Fig. 2B), but their size differed significantly from those of the wild type (Fig. 3, Table 1). Most notably, lodicule depth was significantly reduced in all three mutants, being reduced most in *AP2-D2* (Fig. 3C). Length and width showed a similar tendency in *AP2-D2*, though not in *AP2-A1* or *AP2-D1* (Fig. 3A, 3B).

**Fig. 4.**
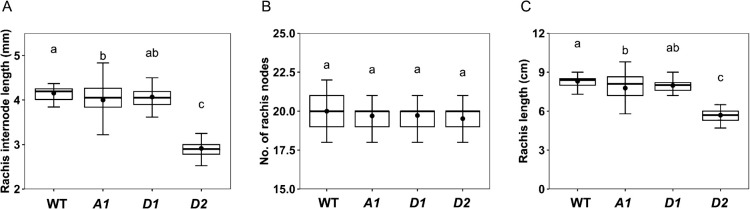
Comparison of spike rachis traits among wild-type (WT) and *AP2* mutants (*A1*, *D1*, *D2*): (A) Rachis internode length. (B) Number of rachis nodes. (C) Rachis length. Thick horizontal lines indicate median (50% quartile), bullets indicate mean, and whiskers represent maximum and minimum values excluding outliers. Mean values with the same letter do not differ significantly (*P* > 0.05) by Tukey–Kramer HSD test.

*AP2-D2* had much shorter spikes than the other mutants (see rachis length in Fig. 4C, Table 1) and showed a typical compact (dense) spike (Fig. 2A). The shorter spike of *AP2-D2* was caused by a significant reduction in rachis internode length (Fig. 4A, Table 1), not in the number of rachis nodes (Fig. 4B). The decrease in length was also found in *AP2-A1* and *AP2-D1* mutants, although to a lesser extent (Fig. 4A).

All three null mutants in which individual *AP2* homoeologs were deleted flowered at anthesis with lodicules swelled. No phenotypes clearly associated with a specific null mutant were found. However, plants showing more general mutational signatures—e.g., poor growth, low fertility, morphological abnormality, etc.—were segregated frequently in the progeny of null mutants. This is likely due to deleterious mutations other than a single deletion of the target *AP2* homoeolog caused by gamma-irradiation. Therefore, we consider that further trait analysis of the null mutants would be inappropriate, and that near-isogenic lines should be used for more precise trait evaluation.

### Transcriptional profiling of mutant alleles

**Fig. 5.**
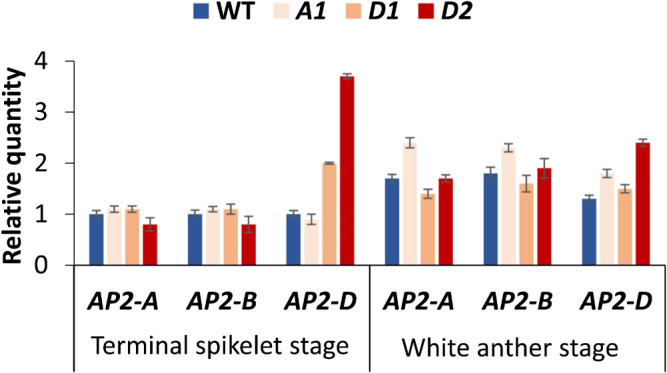
Transcript abundance of *AP2* homoeologs (*AP2-A*, *-B*, *-D*) in immature spikes at terminal spikelet and white anther stages by qPCR assay. Transcript abundances were normalized against that of *actin* and that of the WT at the terminal spikelet stage.

qPCR assay of immature spikes at the terminal spikelet stage and white anther stage revealed that the abundance of the *AP2-D* transcript was remarkably higher in *AP2-D2*, followed by *AP2-D1*, than in the wild type and *AP2-A1*, notably so in the younger spikes at the terminal spikelet stage (Fig. 5). In contrast, the abundance of the *AP2-A* transcript was slightly higher in *AP2-A1* than in the others, especially at the white anther stage, although the differences were not as high as those of the *AP2-D* transcript.

### Predicted interaction between miR172 and the target sites of mutant alleles

**Fig. 6.**
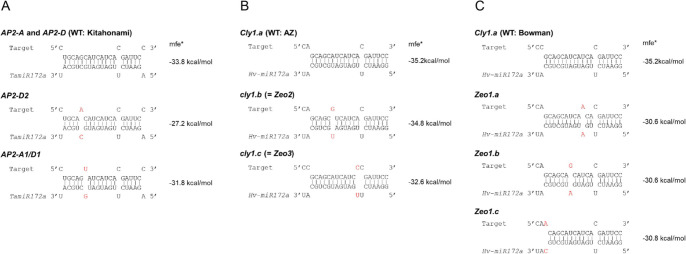
Predicted interactions between mRNA and miR172. (A) Wheat *AP2* homoeologs (this study). (B) Barley *cly1* (Nair *et al.* 2010). (C) Barley *Zeo1* (Houston *et al.* 2013). *mfe: minimum free energy (Rehmsmeier *et al.* 2004).

Relative to that in the wild type (mfe = –33.8 kcal/mol), the predicted interactions between miR172 and the target sites were reduced in all three mutants, more so in *AP2-D2* (mfe = –27.2 kcal/mol) than in *AP2-A1* and *AP2-D1* (mfe = –31.8 kcal/mol; Fig. 6A). The result suggests that reduced interaction between mRNA and miR172 at the target site suppresses lodicule development and leads to a compact spike, as supported by similar results in barley (Fig. 6B, 6C; described further in the Discussion).

## Discussion

### Point mutations in the miR172 target site inhibit lodicule development

Lodicule swelling is the main factor that opens flowers at anthesis in wheat and other cereals by pushing apart the lemma and palea (Nair *et al.* 2010, Ning *et al.* 2013a, Ohmori *et al.* 2018, Yoshida *et al.* 2007). We found that lodicule size, particularly depth, was significantly reduced in all three mutants carrying a point mutation of wheat *AP2* homoeologs. However, none of the mutants flowered closed. This result indicates that the lodicules of all mutants could swell and expand, not fully but enough for floret opening, despite a universal mechanical function of lodicule depth subject to SNPs in the miR172 target site in barley and wheat. The greatest reduction in lodicule depth was seen in the *AP2-D2* mutant, indicating that this mutation had larger functional effects than the other two mutations. We presume that the larger effect was caused by the reduced interaction between miR172 and the target mRNA sites of the mutants, described next.

### Reduced interaction of miR172-mediated mRNA cleavage reduces lodicule size

Base pairing between miRNA and its target mRNA is crucial for mRNA cleavage (Huntzinger and Izaurralde 2011). Differences in miR172-guided cleavage of *Cly1* transcripts alter lodicule development and the consequent occurrence of cleistogamy (Nair *et al.* 2010). Importantly, similar mutations replace a strong G:C pair in the wild type with a G:U wobble pair (Rehmsmeier *et al.* 2004, Varani and McClain 2000) in *AP2-A1* and *AP2-D1*, which could maintain weak interactions. An A:C mismatch reduces the interaction with miR172 much more in *AP2-D2* than in *AP2-A1* and *AP2-D1*. The differences in interactions with miR172 could also affect the amount of AP2 protein translated, which is necessary for suppressing the development of lodicules.

The pronounced effect of the *AP2-D2* allele on lodicule size can be attributed to its significantly lower interaction with miR172 at the target site. This reduces the likelihood of miR172-mediated mRNA cleavage, resulting in higher transcript levels than from the *AP2-A1*, *AP2-D1*, and wild-type alleles. The results of qPCR analysis support this, showing high *AP2-D2* transcript levels at both spike development stages (Fig. 5). Moreover, all SNP mutants expressed the gene at both development stages, *AP2-D2* particularly so. A previous expression analysis in barley revealed that, while the expression patterns of *Cly1.a* (cv. Azumamugi, AZ) and *cly1.b* (cv. Kanto Nakate Gold, KNG) are similar from the awn primordium stage to the yellow anther stage, the transcript abundance of KNG is higher (Nair *et al.* 2010). miR172a regulates the expression of *Cly1* by inhibiting its translation, and the level of Cly1 protein is reduced in AZ (Anwar *et al.* 2018). It can be inferred that the abundance of translated protein in *AP2-D2* was sufficient to inhibit the full expansion of lodicules, although the difference in amino acid change between *AP2-D2* and *AP2-A1*/*D1* might also contribute to the differential reduction in lodicule size.

### Reduced interaction between miRNA and its target mRNA at the miR172 target site results in compact spike morphology

Barley’s cleistogamy alleles (*cly1.b* and *cly1.c*) derive from natural *HvAP2* gene variants (Nair *et al.* 2010). Induced *Zeocriton* (*Zeo*) gene mutants had interesting mutations in *HvAP2*, with three different point mutations identified in the miR172 target site (Houston *et al.* 2013, Fig. 6C). The *Zeo1* mutant showed a very compact spike morphology with a remarkable increase in spike density. The phenotype was caused by significantly reduced elongation of rachis internodes. This dense-spike phenotype was much more severe in *Zeo1* than in *Zeo2* (=*cly1.b*) and *Zeo3* (=*cly1.c*). The degree of interaction with miR172 was predicted to be reduced relative to that of the wild-type allele (*Cly1.a*, mfe = –35.2 kcal/mol) to a much greater extent in *Zeo1* alleles (mfe = –30.6 to –30.8 kcal/mol) than in *Zeo2/cly1.b* (mfe = –34.8 kcal/mol) or *Zeo3*/*cly1.c* (mfe = –32.6 kcal/mol; Fig. 6B, 6C). Therefore, its severity in *Zeo1* may be due to the greater reduction in the miR172 binding affinity. Our result of wheat *AP2* mRNA interaction with miR172 supports this, where *AP2-D2* had a lower interaction than *AP2-A1* and *AP2-D1* (Fig. 6A), resulting in a dense-spike morphology. Higher transcript levels result in a compact spike and reduced plant height, as found in another wheat *AP2* gene, *Q* (Simons *et al.* 2006). The result shows that the effect of the SNP in the miR172 target site on the reduction of rachis internode length is common in barley and wheat. Furthermore, the *Zeo1* mutants flower cleistogamously and lack lodicule swelling at anthesis.

### Concluding remarks

This study evaluated the potential for developing cleistogamous wheat by analyzing three novel mutant alleles of *AP2* homoeologs. Lodicule depth was reduced in the *AP2-A1*, *AP2-D1*, and *AP2-D2* mutants, although cleistogamy as observed in barley mutants was not induced. Although the mechanism is not elucidated, the chasmogamous phenotype is expressed in barley genotypes heterozygous for *cly1* (*Cly1.a/cly1.b*) (Nair *et al.* 2010). In analogy with this phenomenon, the two wild-type homoeologs may compensate the effect of one mutated homoeolog in hexaploid wheat. Consequently, it would be interesting to test the combination of two mutant homoeologs along with the null mutants to investigate dosage effects of *AP2* mutations on cleistogamy in wheat. Targeted mutagenesis of *AP2-B* homoeologs may also be needed to test the initial hypothesis that combining miRNA target site mutations in all three *AP2* homoeologs within a single plant would result in the production of cleistogamous wheat (Ning *et al.* 2013a). In addition to flowering traits, pleiotropy of *AP2* mutations is of particular importance, as it might influence a wide range of agronomic traits as well as spike density. Trait evaluation in a near-isogenic genetic background will be required for more precise evaluation of quantitative traits. All these issues need to be addressed in the next step of this research.

## Author Contribution Statement

TK and KK designed the study. KS, FK, YO, and HH conducted mutagenesis, generation of mutant populations, and screening of mutants. ABN, HMH, and KK performed the field survey and molecular experiments on mutants, and analyzed the data. ABN, TK, and KK wrote the manuscript.

## Supplementary Material

Supplemental Figures

Supplemental Table

## Figures and Tables

**Table 1.** Comparison of lodicule size and spike traits among the wild type and three *AP2* mutants (mean ± S.E.)

Genotype	Lodicule size					Spike traits			
	*n*	Length (mm)	Width (mm)	Depth (mm)		*n*	Rachis internode length (mm)	Rachis node number	Rachis length (cm)
WT	32	1.69 ± 0.02	0.90 ± 0.02	1.15 ± 0.02		30	4.15 ± 0.03	20.0 ± 0.17	8.30 ± 0.08
*A1*	32	1.62 ± 0.02	0.90 ± 0.01	1.02 ± 0.01		63	4.00 ± 0.05	19.7 ± 0.13	7.77 ± 0.17
*D1*	30	1.67 ± 0.02	0.94 ± 0.02	1.05 ± 0.02		69	4.07 ± 0.02	19.7 ± 0.09	7.98 ± 0.06
*D2*	26	1.49 ± 0.02	0.86 ± 0.02	0.92 ± 0.01		75	2.91 ± 0.02	19.5 ± 0.11	5.69 ± 0.06
ANOVA		***	*	***			***	n.s.	***

*, *** and n.s.: significant at 5%, 0.1% and not significant at 5% levels, respectively. *n*: number of lodicules and spikes used.

## References

[B1] Anwar, N., M. Ohta, T. Yazawa, Y. Sato, C. Li, A. Tagiri, M. Sakuma, T. Nussbaumer, P. Bregitzer, M. Pourkheirandish et al. (2018) miR172 downregulates the translation of *cleistogamy 1* in barley. Ann Bot 122: 251–265. 29790929 10.1093/aob/mcy058 PMC6070043

[B2] Houston, K., S.M. McKim, J. Comadran, N. Bonar, I. Druka, N. Uzrek, E. Cirillo, J. Guzy-Wrobelska, N.C. Collins, C. Halpin et al. (2013) Variation in the interaction between alleles of *HvAPETALA2* and microRNA172 determines the density of grains on the barley inflorescence. Proc Natl Acad Sci USA 110: 16675–16680. 24065816 10.1073/pnas.1311681110 PMC3799380

[B3] Huntzinger, E. and E. Izaurralde (2011) Gene silencing by microRNAs: contributions of translational repression and mRNA decay. Nat Rev Genet 12: 99–110. 21245828 10.1038/nrg2936

[B4] Kirby, E.J.M. and M. Appleyard (1981) Cereal development guide. National Agricultural Centre Cereal Unit, Stoneleigh, Warwickshire, UK.

[B5] Komatsuda, T., I. Nakamura, F. Takaiwa and S. Oka (1998) Development of STS markers closely linked to the *vrs1* locus in barley, *Hordeum vulgare*. Genome 41: 680–685.

[B6] Komura, S., H. Jinno, T. Sonoda, Y. Oono, H. Handa, S. Takumi, K. Yoshida and F. Kobayashi (2022) Genome sequencing-based coverage analyses facilitate high-resolution detection of deletions linked to phenotypes of gamma-irradiated wheat mutants. BMC Genomics 23: 111. 35139819 10.1186/s12864-022-08344-8 PMC8827196

[B7] Livak, K.J. and T.D. Schmittgen (2001) Analysis of relative gene expression data using real-time quantitative PCR and the 2^–ΔΔ^C_T_ method. Methods 25: 402–408. 11846609 10.1006/meth.2001.1262

[B8] Nair, S.K., N. Wang, Y. Turuspekov, M. Pourkheirandish, S. Sinsuwongwat, G. Chen, M. Sameri, A. Tagiri, I. Honda, Y. Watanabe et al. (2010) Cleistogamous flowering in barley arises from the suppression of microRNA-guided *HvAP2* mRNA cleavage. Proc Natl Acad Sci USA 107: 490–495. 20018663 10.1073/pnas.0909097107 PMC2806734

[B9] Ning, S., N. Wang, S. Sakuma, M. Pourkheirandish, J. Wu, T. Matsumoto, T. Koba and T. Komatsuda (2013a) Structure, transcription and post-transcriptional regulation of the bread wheat orthologs of the barley cleistogamy gene *Cly1*. Theor Appl Genet 126: 1273–1283. 23381807 10.1007/s00122-013-2052-6

[B10] Ning, S., N. Wang, S. Sakuma, M. Pourkheirandish, T. Koba and T. Komatsuda (2013b) Variation in the wheat *AP2* homoeologs, the genes underlying lodicule development. Breed Sci 63: 255–266. 24273420 10.1270/jsbbs.63.255 PMC3770552

[B11] Ohmori, S., S. Koike, T. Hayashi, T. Yamaguchi, M. Kuroki and H. Yoshida (2018) The cleistogamy of the *superwoman1-cleistogamy1* mutation is sensitive to low temperatures during the lodicule-forming stage. Breed Sci 68: 432–441. 30369817 10.1270/jsbbs.18028 PMC6198900

[B12] Rehmsmeier, M., P. Steffen, M. Höchsmann and R. Giegerich (2004) Fast and effective prediction of microRNA/target duplexes. RNA 10: 1507–1517. 15383676 10.1261/rna.5248604 PMC1370637

[B13] Simons, K.J., J.P. Fellers, H.N. Trick, Z. Zhang, Y.S. Tai, B.S. Gill and J.D. Faris (2006) Molecular characterization of the major wheat domestication gene *Q*. Genetics 172: 547–555. 16172507 10.1534/genetics.105.044727 PMC1456182

[B14] Turuspekov, Y., Y. Mano, I. Honda, N. Kawada, Y. Watanabe and T. Komatsuda (2004) Identification and mapping of cleistogamy genes in barley. Theor Appl Genet 109: 480–487. 15138690 10.1007/s00122-004-1673-1

[B15] Varani, G. and W.H. McClain (2000) The G·U wobble base pair: A fundamental building block of RNA structure crucial to RNA function in diverse biological systems. EMBO Rep 1: 18–23. 11256617 10.1093/embo-reports/kvd001 PMC1083677

[B16] Wang, N., K. Kakeda, M. Tomokazu, C. Liu, M. Yoshida, N. Kawada and T. Komatsuda (2021) A novel mutant allele at the *Cleistogamy 1* locus in barley. Theor Appl Genet 134: 3183–3193. 34125245 10.1007/s00122-021-03884-1

[B17] Yoshida, M., N. Kawada and T. Tohnooka (2005) Effect of row type, flowering type and several other spike characters on resistance to Fusarium head blight in barley. Euphytica 141: 217–227.

[B18] Yoshida, M., N. Kawada and T. Nakajima (2007) Effect of infection timing on fusarium head blight and mycotoxin accumulation in open- and closed-flowering barley. Phytopathology 97: 1054–1062. 18944170 10.1094/PHYTO-97-9-1054

