# Supplementary material for: Mutations within the miR172 target site of wheat AP2 homoeologs regulate lodicule size and rachis internode length
Source: Breed Sci. 2023 Sep 9;73(4):401–7. doi: 10.1270/jsbbs.23019 (PMC10722097; doi:10.1270/jsbbs.23019)
Supplement: Supplementary file 1 — Supplemental Figures [file 73_401_s1.pdf]

doi: 10.1270/jsbbs.23019

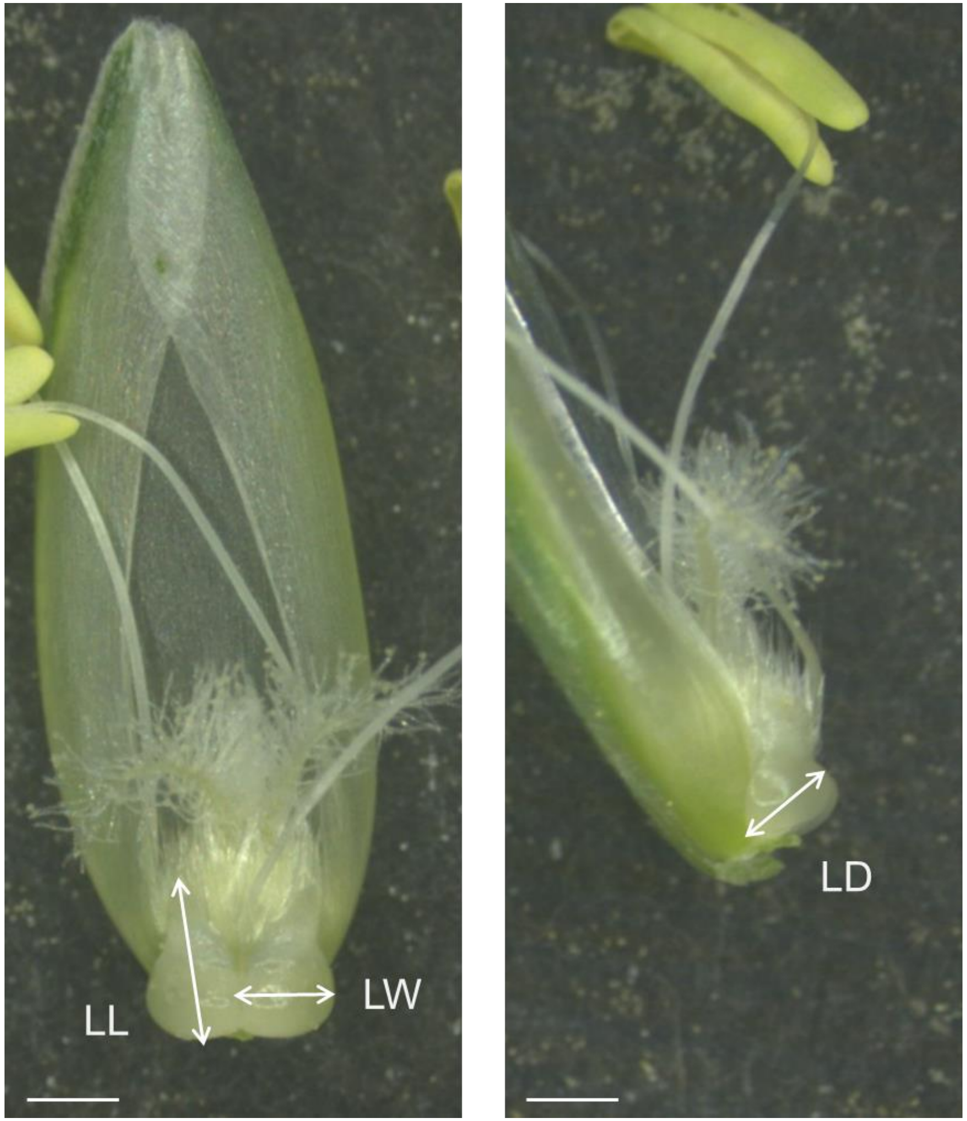

Supplemental Fig. 1.
Measurement of lodicule length (LL), width (LW), and depth (LD). Bar, 1 mm.

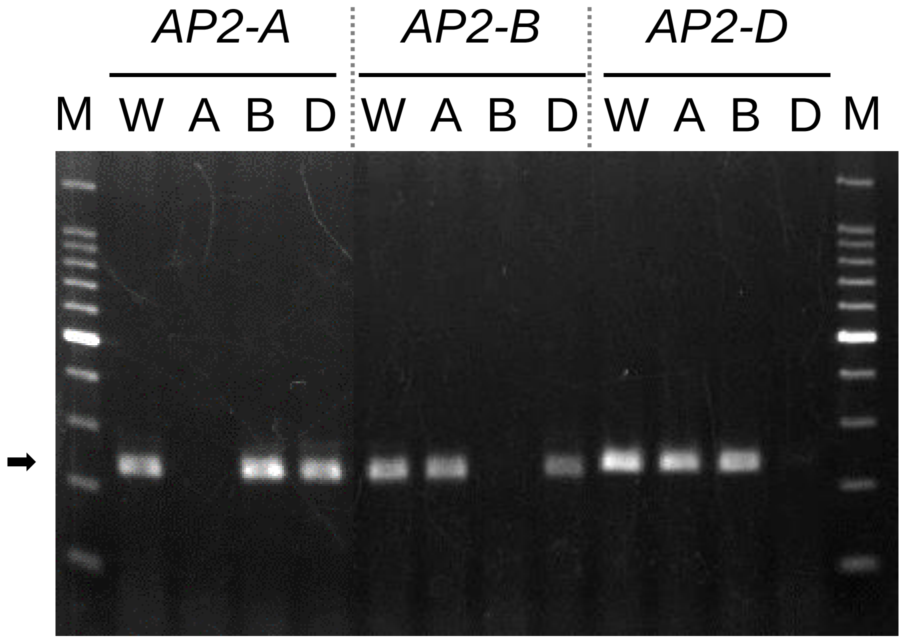

W : WT (‘Kitahonami’)
A : Null mutant for *AP2-A* (1131A)
B : Null mutant for *AP2-B* (169B)
D : Null mutant for *AP2-D* (248D)
M : Molecular weight marker

Supplemental Fig. 2.
Gene-specific PCR showing the null (deletion) mutation in each *AP2* homoeolog. *AP2-A*, *AP2-B*, and *AP2-D* genes are deleted in the null mutants 1131A, 169B, and 248D, respectively. An arrow indicates the amplified product of target gene.
