# Supplementary material for: Mutations within the miR172 target site of wheat AP2 homoeologs regulate lodicule size and rachis internode length
Source: Breed Sci. 2023 Sep 9;73(4):401–7. doi: 10.1270/jsbbs.23019 (PMC10722097; doi:10.1270/jsbbs.23019)
Supplement: Supplementary file 2 — Supplemental Table [file 73_401_s2.pdf]

doi: 10.1270/jsbbs.23019

Supplemental Table 1. Primer sequences used in this study

Category	Target gene	Primer name	Primer sequence (5'-3')	Primer name	Primer sequence (5'-3')
Gene-specific amplification	*AP2-A*	F-est1320	TGCACGGCTGGGGCAACGTC	L3721A19	CGGTGGTGGAGCTGGCAAG
(HRM and null mutant screening)	*AP2-B*	F-est1180	TCATGGGCAACGGTGATCC	L3801L18	GCGCTGGCTGCTCTCGAC
	AP2-D	F-est1320	TGCACGGCTGGGGCAACGTC	L3897D22	GGTGGAGCTGGTCTTGATGGTC
3' UTR for qPCR	*AP2-A*	A3718U19	AAGCTTGCCAGCTCCACCA	A3794L19	CCCATGCTCCTCCGTGATC
(Real-time PCR)	*AP2-B*	B3960U19	GAGCTGAACTGAAACTTGA	B4052L19	CCGCATTTACATGACTGCC
	AP2-D	D3897U20	GACCATCAAGACCAGCTCCA	D4007L18	TGCATGCATGGTTGTGGT
	Actin	DN182500F425	AAGTACAGTGTCTGGATTGGAGGG	DN182500R533	TCGCAACTTAGAAGCACTTCCG
